# Lipid metabolism: a potential checkpoint for cGAS-STING regulation in colorectal cancer

**DOI:** 10.3389/fimmu.2026.1928380

**Published:** 2026-10-05

**Authors:** Qi Qiu, Zhihong Cheng, Jian Gu, Tao Zhang, Mingdong Cai, Jiliang Wang

**Affiliations:** 1 Department of Gastrointestinal Surgery, Union Hospital, Tongji Medical College, Huazhong University of Science and Technology, Wuhan, China; 2 Hubei Provincial Clinical Research Center for Minimally Invasive Surgery, Union Hospital, Tongji Medical College, Huazhong University of Science and Technology, Wuhan, China; 3 Department of Anesthesiology, Union Hospital, Tongji Medical College, Huazhong University of Science and Technology, Wuhan, China; 4 Institute of Anesthesia and Critical Care Medicine, Union Hospital, Tongji Medical College, Huazhong University of Science and Technology, Wuhan, China

**Keywords:** cGAS-STING pathway, colorectal cancer, immunotherapy, lipid metabolism reprogramming, metabolic-immune crosstalk

## Abstract

Immunotherapy for colorectal cancer (CRC) continues to face significant challenges, particularly in patients with microsatellite stable (MSS) tumors. The cyclic GMP–AMP synthase–stimulator of interferon genes (cGAS–STING) pathway is a key regulator of innate immunity and has emerged as a promising target for enhancing anti-tumor immune responses. Meanwhile, lipid metabolic reprogramming is a defining hallmark of CRC and contributes to tumor progression, evasion, and therapeutic resistance. Although direct evidence linking lipid metabolism to cGAS–STING signaling in CRC remains limited, accumulating studies from diverse disease models suggest extensive interactions between these two processes. This review summarizes the major features of lipid metabolic reprogramming in CRC and discusses current understanding of cGAS–STING signaling in tumor immunity. We further examine potential mechanisms by which lipid metabolism may influence cGAS–STING activity, including the regulation of mitochondrial DNA release, intracellular trafficking of signaling components and lipid-associated post-translational modifications. Emerging evidence also indicates that cGAS–STING signaling can affect lipid metabolic pathways by regulating lipid metabolic homeostasis and inducing lipid peroxidation-driven ferroptosis. Importantly, microbiota-derived lipid metabolites exert context-dependent effects on cGAS–STING signaling to balance intestinal inflammation and anti-tumor immunity. Finally, we discuss therapeutic strategies targeting lipid metabolism and cGAS–STING signaling, with a particular focus on approaches that may enhance anti-tumor immunity through coordinated metabolic-immune modulation. By bridging metabolic reprogramming with innate immune sensing, we propose that modulating the lipid-cGAS-STING axis could break immune tolerance and offer a distinct therapeutic window for CRC.

## 1 Introduction

Colorectal cancer (CRC) ranks as the third most prevalent malignancy and the second leading cause of cancer-related mortality worldwide, creating a severe burden on global public health (1, 2). Although immune checkpoint inhibitor (ICI) therapy has demonstrated great potential in oncology, its efficacy in CRC varies substantially across patients. Only approximately 15% of patients with microsatellite instability-high (MSI-H) tumors derive clinical benefit, whereas the vast majority—those with microsatellite stable (MSS) tumors—are largely refractory to single-agent immunotherapy, typically presenting with an “immune desert” or “immune-excluded” phenotype (3). Therefore, remodeling the immune microenvironment of MSS CRC to convert these tumors from a “cold” to a “hot” state represents an urgent clinical need.

The cyclic GMP-AMP synthase (cGAS)-stimulator of interferon genes (STING) signaling pathway links innate and adaptive immunity and is pivotal in shifting the tumor immune microenvironment (TIME) from “cold” to “hot” (4). However, recent clinical trials have indicated that monotherapies targeting STING yield limited efficacy, implying the existence of a more intricate regulatory network in CRC.

Metabolic reprogramming is recognized as one of the core hallmarks of cancer (5). Particularly, dysregulated lipid metabolism, often reflected by obesity and metabolic syndrome, is strongly linked to an increased risk of CRC (6). To sustain their rapid proliferation and invasion, CRC cells frequently rewire lipid uptake, synthesis, and catabolism (7). Crucially, lipids actively participate in regulating cGAS-STING signaling. For instance, STING activation requires specific lipid modifications such as palmitoylation (8, 9). Consequently, aberrant lipid metabolism likely influences cGAS-STING function. Yet, the specific crosstalk between these processes in CRC remains poorly understood.

Given the recognized impact of lipid metabolic reprogramming in CRC, uncovering its mechanisms for regulating cGAS-STING could provide new strategies to overcome the “cold” immune microenvironment. In this review, we summarize the recent advances regarding lipid metabolism and cGAS-STING in CRC, explore their underlying interaction mechanisms, and propose a hypothetical framework for their crosstalk (**Figure 1**).

**Figure 1:**
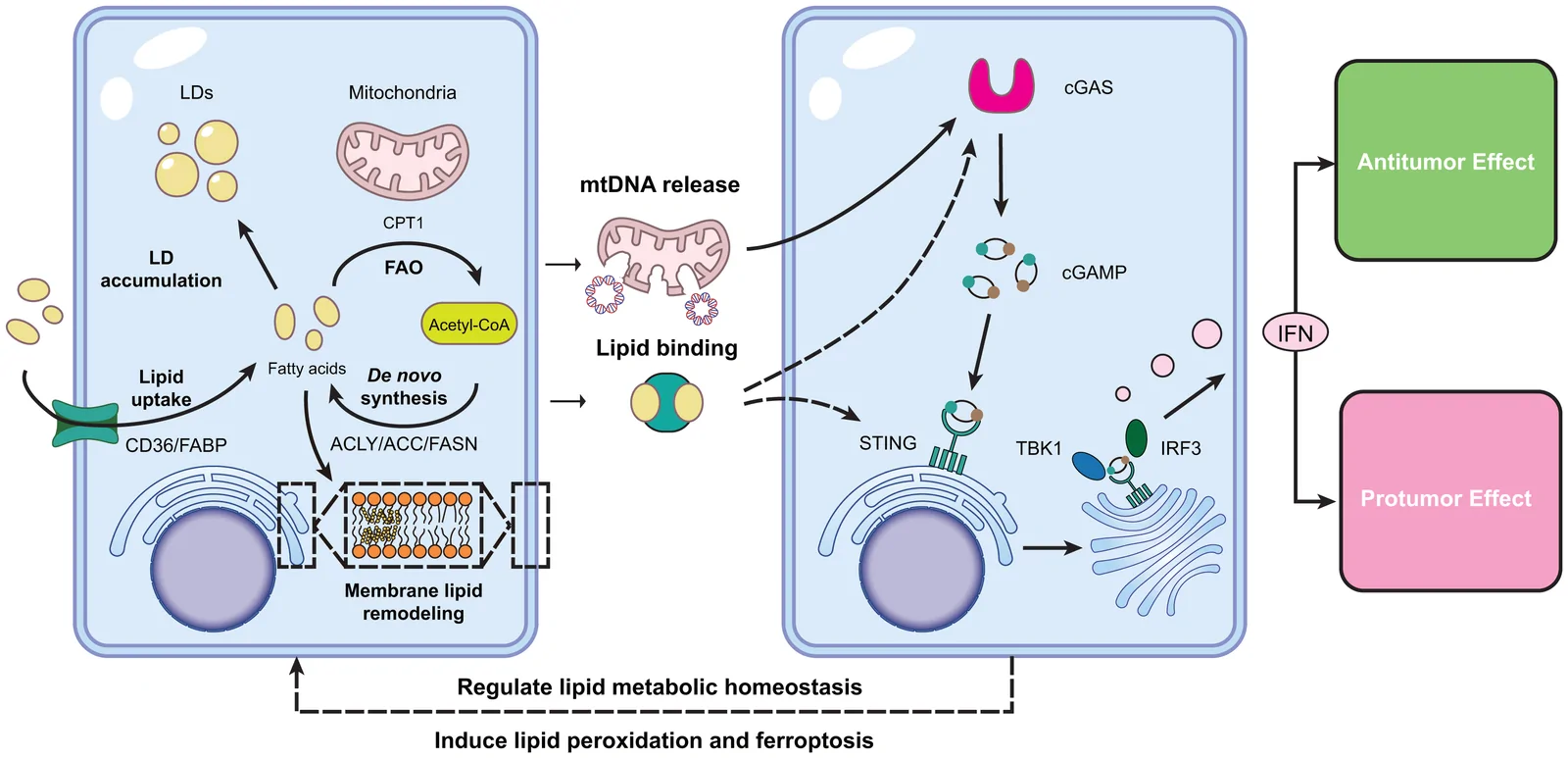
The crosstalk between lipid metabolic reprogramming and the cGAS-STING pathway in colorectal cancer. Lipid Metabolic Reprogramming (Left Panel): CRC cells undergo extensive lipid metabolic reprogramming. This involves enhanced exogenous lipid uptake mediated by CD36 and FABPs, and increased *de novo* fatty acid synthesis driven by the ACLY/ACC/FASN enzymatic cascade. Additionally, tumor cells upregulate FAO via CPT1 in the mitochondria to maintain energy balance, promote LD accumulation as a metabolic buffer, and execute membrane lipid remodeling. The cGAS-STING Pathway (Right Panel): The cGAS-STING pathway is primarily triggered by the release of abnormal DNA, including mtDNA, into the cytosol. Cytosolic cGAS recognizes mtDNA and catalyzes the synthesis of the second messenger cGAMP. cGAMP then binds to and activates STING, which recruits and activates TBK1 and IRF3 to drive interferon (IFN) secretion. In CRC, downstream IFN signaling is highly context-dependent, culminating in either antitumor immunity or protumor effects. Metabolic-Immune Interplay (Middle and Bottom): A complex, bidirectional regulatory network exists between these two systems. Aberrant FAO and LD accumulation can disrupt mitochondrial integrity, affecting mtDNA release to activate cGAS. Enhanced lipid uptake and *de novo* synthesis lead to lipid accumulation, which subsequently regulates subcellular trafficking and activation of cGAS-STING components through direct lipid binding and altered lipid microenvironments. Conversely, the activation of the cGAS-STING pathway inversely reshapes lipid metabolism by regulating lipid metabolic homeostasis and inducing lipid peroxidation-driven ferroptosis. Solid lines indicate associations validated in colorectal cancer (CRC), whereas dashed lines represent those established in other diseases. CRC, colorectal cancer; FABPs, fatty acid-binding proteins; ACLY, ATP citrate lyase; ACC, acetyl-CoA carboxylase; FASN, fatty acid synthase; FAO, fatty acid oxidation; CPT1, carnitine palmitoyltransferase 1; LDs, lipid droplets; mtDNA, mitochondrial DNA; cGAS, cyclic GMP-AMP synthase; cGAMP, cyclic guanosine monophosphate-adenosine monophosphate; STING, stimulator of interferon genes; TBK1, TANK-binding kinase 1; IRF3, interferon regulatory factor 3; IFN, interferon.

## 2 CRC cells and lipid metabolic reprogramming

Lipid metabolism involves processes such as lipid uptake, synthesis, oxidation, and storage. To adapt to the hostile environment of hypoxia, nutrient deprivation, and immune surveillance, CRC cells actively reprogram these metabolic pathways, providing the material basis for their proliferation, invasion, and immune evasion.

### 2.1 Enhanced exogenous fatty acid uptake and *de novo* synthesis

Lipids are fundamental for cellular proliferation and signal transduction, and enhanced *de novo* fatty acid synthesis is a metabolic hallmark of malignancy (10). In CRC, the enzymatic cascade comprising ATP citrate lyase (ACLY), acetyl-CoA carboxylase(ACC), and fatty acid synthase (FASN)—which sequentially converts acetyl-CoA into saturated fatty acids—is frequently hyperactivated and correlates with poor prognosis. Mechanistically, ACLY has been shown to enhance CRC invasion, migration, and chemoresistance partially by stabilizing and supporting the nuclear translocation of β-catenin (11, 12). Transcriptional activation of *ACC1* promotes tumor growth and epithelial-mesenchymal transition (EMT) (13), while *FASN* is governed by oncogenic signals to drive cancer progression (14). Sterol regulatory element-binding protein 1 (SREBP1) orchestrates this lipogenic cascade to maintain tumor stemness (15). Therefore, targeting *de novo* lipogenesis presents a promising therapeutic vulnerability. For example, targeting a specific long non-coding RNA can limit ACC activity to promote ferroptosis in CRC cells (16).

Beyond *de novo* synthesis, CRC cells acquire lipids via exogenous uptake. A major fatty acid transporter, CD36, is upregulated in CRC, particularly in metastatic lesions, indicating a heightened dependency on external lipids during dissemination (17). CD36 interacts with perilipin 2 (PLIN2) to drive EMT (18), although some studies suggest it may delay CRC progression by suppressing glycolysis (19), necessitating further investigation. Fatty acid-binding proteins (FABPs) are also implicated in CRC progression. FABP4 promotes EMT by downregulating E-cadherin and upregulating Snail (20, 21), whereas FABP6 mediates bile acid transport during early carcinogenesis (22, 23). Prognostic model analysis revealed that CD36 and FABP4 expression is independently associated with poor prognosis in patients with MSS CRC (24). Notably, FASN inhibition induces a compensatory upregulation of CD36, underscoring a dynamic synergy between lipid synthesis and uptake to sustain tumor survival (25).

Lipid metabolic reprogramming modulates the crosstalk between CRC cells and the tumor microenvironment (TME). CRC cells directly internalize adipocyte-derived fatty acids for β-oxidation (26) and secrete insulin-like growth factor-binding protein (IGFBP3) to induce lipolysis in surrounding adipocytes, actively remodeling the TME to fulfill their metabolic demands (27). More prominently, enhanced exogenous fatty acid uptake and *de novo* synthesis lead to a highly lipid-enriched TME, which may induce metabolic lipotoxicity and functional exhaustion in infiltrating anti-tumor immune cells (28) and enhance the capacity of immunosuppressive populations (29). Even when anti-tumor immune pathways are activated, their effector functions are blunted. This complex network further underscores the pivotal role of lipid metabolism in CRC progression and immune evasion.

### 2.2 Fatty acid oxidation and metabolic plasticity

Alongside enhanced lipid synthesis and uptake, CRC cells frequently exhibit elevated fatty acid oxidation (FAO). Activated FAO pathway not only supplies ATP but also generates reducing equivalents (NADPH) to maintain redox homeostasis and counteract reactive oxygen species (ROS), which fuels anoikis resistance in metastatic CRC cells (30). Carnitine palmitoyltransferase 1A (CPT1A), the rate-limiting enzyme of FAO, is significantly upregulated in CRC and correlates with reduced median patient survival (31). CPT1A expression is further enriched in metastatic lesions (30), likely because disseminating CRC cells use valosin-containing protein (VCP) to degrade histone deacetylase 1 (HDAC1), which transactivates CPT1A to overcome the stress of glucose deprivation (32). Furthermore, CPT1A suppresses apoptosis by modulating mitochondrial permeability (31). Enhanced FAO also helps shape other malignant phenotypes. Ring finger protein 183 (RNF183) preserves tumor stemness by bolstering FAO (33). Consistent with observations in other malignancies, isolated FAO inhibition triggers a compensatory glycolytic surge and drug resistance (34). Conversely, dual blockade of glycolysis and FAO exerts synergistic anti-tumor efficacy in CRC (32), underscoring FAO’s compensatory role within the tumor’s energetic network. In conclusion, CRC cells maintain a dynamic equilibrium between lipid utilization, anabolism, and global energy metabolism, reflecting metabolic plasticity through regulating FAO.

### 2.3 Lipid storage and buffering systems

As FAO is often insufficient to catabolize the increased lipid influx in CRC cells, they activate a metabolic buffering system centered on lipid droplets (LDs). Abundant LD accumulation is a key hallmark of invasiveness and therapeutic resistance in CRC stem cells (35). In addition, the lipid droplet-associated proteins PLIN1 and PLIN4 offer complementary prognostic information in MSS CRC (24). LDs serve not only as energy reservoirs for tumor growth and metastasis but also act as crucial shields against oxidative stress and lipotoxicity. By rapidly esterifying and sequestering excess free fatty acids (FFAs), LDs prevent FFA overload-induced endoplasmic reticulum (ER) stress and mitochondrial damage (36, 37). Through these protective mechanisms, aberrant LD accumulation suppresses immunogenic cell death, dampens anti-tumor immune responses within the microenvironment, and confers chemoresistance (38, 39). As independent hydrophobic organelles, LDs can also directly sequester specific lipophilic cytotoxic drugs within their cores, physically isolating them from their intracellular targets (40). Overall, LD accumulation represents an adaptive mechanism enabling CRC cells to tolerate metabolic stress and cytotoxicity.

### 2.4 Phospholipid/Cholesterol homeostasis and membrane lipid remodeling

Biological membranes primarily consist of phospholipids and cholesterol. Through metabolic reprogramming, CRC cells specifically remodel these components to alter plasma membrane physicochemistry, constructing an adaptive barrier that facilitates malignant proliferation, drug resistance, and stress survival.

Membrane phospholipids exhibit marked desaturation in CRC. Tumor cells convert saturated fatty acids (SFAs) into monounsaturated fatty acids (MUFAs) and polyunsaturated fatty acids (PUFAs) by upregulating stearoyl-CoA desaturase 1 (SCD1) and fatty acid desaturase 2 (FADS2). Mass spectrometry imaging and lipidomic analyses have confirmed that the proportion of unsaturated fatty acids is significantly increased in CRC tissues (41, 42). This desaturation markedly enhances plasma membrane fluidity, making CRC cells more adaptable to mechanical stress induced by microtubule inhibitors such as docetaxel, thereby conferring drug resistance (43). Moreover, an appropriate MUFA/PUFA ratio permits only a controlled, limited increase in ROS-susceptible substrates, limiting lipid peroxidation and protecting CRC cells against ferroptosis and oxidative cytotoxicity.

Aberrant cholesterol metabolism further regulates membrane dynamics. Under the regulation of the sterol regulatory element-binding protein (SREBP2), cholesterol synthesis in CRC cells is significantly enhanced. At the cell membrane level, excess cholesterol is embedded in the lipid bilayer, reducing membrane permeability and the passive diffusion rate of chemotherapeutic drugs such as cisplatin (44). At the same time, cholesterol helps regulate the spatial conformation of drug resistance-related transporters such as P-gp (45) and MRP4 (46), promoting the excretion of toxic metabolites and antitumor drugs and thereby enhancing the multidrug resistance phenotype of tumors (47). Furthermore, cholesterol is not uniformly embedded in the membrane; part of it co-accumulates with sphingolipids to form lipid rafts, which serve as efficient signal transduction platforms (48). These lipid rafts are enriched with various pro-oncogenic effector molecules, lowering the activation energy of enzymatic reactions while spatially segregating negative regulatory factors located outside the raft regions (49). Consequently, weak extracellular stimuli such as those from the Wnt pathway are rapidly amplified, transforming into proliferative and anti-apoptotic signals in CRC cells. At the organelle membrane level, cholesterol enrichment alters ER membrane curvature, potentially impairing the transport efficiency of transmembrane proteins such as STING and dampening downstream antitumor effects (50, 51). Notably, increased cholesterol reduces mitochondrial membrane permeability, attenuating the Bax-mediated apoptotic pathway (52). Additionally, it can induce low-level ROS accumulation, triggering mitohormesis in cells and further enhancing their survival adaptability (47, 53).

In summary, through lipid metabolic reprogramming, CRC cells establish a membrane lipid environment characterized by both a highly fluid matrix with high unsaturated fatty acid composition and rigid microdomains with high cholesterol content, facilitating tumor proliferation and invasion.

## 3 CRC and cGAS-STING pathway

The cGAS-STING pathway is a key cytoplasmic DNA sensing mechanism that plays a critical role in innate immunity (54). cGAS senses cytosolic double-stranded DNA (dsDNA). When abnormal leakage of mitochondrial DNA (mtDNA) or exogenous DNA is detected, cGAS binds to the aberrant dsDNA and catalyzes the synthesis of the second messenger cyclic guanosine monophosphate–adenosine monophosphate (cGAMP). cGAMP binds to and activates the STING protein on the ER, inducing conformational change and oligomerization and triggering translocation to the Golgi apparatus. This subsequently recruits and activates TANK-binding kinase 1 (TBK1). Activated TBK1 phosphorylates interferon regulatory factor 3 (IRF3) and nuclear factor κβ (NF-κβ), promoting the expression and secretion of type I interferons (IFN-I) and inflammatory cytokines, and effectively converting tumor antigens into an innate immune response (54). Recent studies indicate that the intrinsic cGAS-STING pathway in tumor cells plays a complex and dual role in the occurrence, progression, and treatment outcomes of CRC.

### 3.1 cGAS-STING pathway in CRC cells

Tumor-intrinsic cGAS–STING signaling appears to substantially influence clinical outcomes in CRC. Studies have shown that higher STING expression is associated with increased intratumoral CD8^+^ T-cell infiltration, reduced lymphovascular invasion, and longer overall survival and progression-free survival (55, 56). In terms of therapy, the efficacy of chemotherapeutic agents such as 5-fluorouracil (5-FU) is also closely linked to the intrinsic cGAS–STING signaling status of CRC cells (57). The antitumor effects of some certain small-molecule agents are abolished after cGAS or STING knockout (58). Moreover, enforcing STING signaling within MSS/pMMR tumors can potentiate the sensitivity of CRC cells to ICIs (59).

Nevertheless, cGAS–STING signaling in CRC cells is often selectively suppressed. *In vitro* studies have shown that although most CRC cell lines still express downstream STING, they fail to efficiently induce IFN-β and pro-inflammatory cytokines upon exogenous stimulation (60). This may be attributed to the blockade of upstream signaling. Damaged DNA and mtDNA serve as crucial sources for signal activation. Factors such as phosphatidylinositol-3,4,5-triphosphate-dependent Rac exchange factor 2 (PREX2) (61) and Ataxia telangiectasia mutated (ATM) (62) may suppress cGAS–STING activation and immune-cell infiltration by promoting DNA repair. In addition, *KRAS*-driven CRC cells can induce DRP1 phosphorylation, thereby reducing mtDNA abundance and further limiting the availability of cGAS–STING activating ligands (63). As the upstream sensor of the pathway, cGAS is silenced in multiple CRC cell lines due to hypermethylation of its promoter region (60). Transcription of *CGAS* gene is also repressed by high expression of lysine acetyltransferase 6A (64).

It should be noted that the molecular characteristics of CRC cells closely influence the activation state of the pathway. MSI CRC cells usually exhibit stronger cGAS-STING signaling compared to MSS cells (65). This may be due to the accumulation of genomic mutations and continuous production of cytosolic dsDNA resulting from MSI, which maintains a higher baseline signaling activity within the tumor cells. Likewise, treatment with chemotherapeutic agents such as cisplatin and camptothecin can induce DNA damage and impair DNA repair, functionally mimicking the MSI state and resulting in potent pathway activation (66).

### 3.2 Context-dependent role of cGAS-STING pathway in CRC

#### 3.2.1 Tumor-suppressive roles of cGAS-STING in CRC

The cGAS-STING pathway exerts anti-tumor effects in CRC and various other tumor models. It inhibits tumors primarily through two mechanisms: remodeling the TIME and directly inducing tumor cell death.

Activation of the cGAS-STING pathway frequently promotes the infiltration and function of immune effector cells. Tumor-derived DNA and cGAMP can transfer into dendritic cells (DCs) to activate cGAS-STING signaling. This promotes DC maturation and enhances their antigen-presenting capacity (67–69). In metastatic CRC, STING activation also reprograms tumor-associated macrophages (TAMs) into the anti-tumor M1 phenotype (70, 71). The pathway is equally crucial for adaptive immunity. High cGAS-STING expression in CRC cells positively correlates with abundant tumor-infiltrating lymphocytes, indicating an immunologically active microenvironment (65). Following STING agonist treatment, DC antigen presentation drives increased CD8^+^ T cell infiltration into the tumor core, strengthening cellular immunity (56). As for CD4^+^ T cells, STING signaling can promote their differentiation into anti-tumor Th1 and Th9 cells in a cell-intrinsic manner and significantly increase secretion of IFN-γ and IL-9 (72). Recent studies have also shown that IFN-I secreted downstream of STING activation promotes the recruitment of memory B cells by inducing the chemokine CCL19, thereby facilitating the assembly of tertiary lymphoid structures and enhancing humoral immunity (73). Accordingly, in CRC, the cGAS–STING pathway serves as a bridge linking innate and adaptive immunity (74). Recent studies show that agonist-based combination therapies significantly reduce the infiltration of immunosuppressive cells, including MDSCs, TAMs, and Tregs (75–77). Combining cGAS-STING activators with anti-PD-1/PD-L1 therapy often yields stronger anti-tumor effects than monotherapy (58, 75, 78–80), further validating its role in TIME remodeling.

Acute cGAS-STING activation can also induce programmed tumor cell death under certain conditions. For instance, lovastatin efficiently activates STING, triggering apoptosis and alleviating chemoresistance (81). STING can bind to spleen tyrosine kinase to activate the NOD-like receptor protein 3 (NLRP3) inflammasome, inducing tumor cell pyroptosis (82). These pathways eliminate tumor cells while releasing danger signals and antigens, which creates a feed-forward loop: DNA and antigens from dying cells re-activate cGAS-STING signaling in surrounding cells, further stimulating immune recognition.

In addition, the physical barrier of the colorectal epithelium confers extra protective functions on the cGAS-STING pathway. Specifically, cGAS maintains intestinal barrier integrity. Its loss exacerbates inflammation, accelerates epithelial proliferation, and promotes carcinogenesis (83). STING signaling coordinates mucosal wound healing and limits colitis-associated tumorigenesis, partly through modulating the IL-22/IL-22 binding protein axis to maintain intestinal tissue homeostasis (84).

#### 3.2.2 Tumor-promoting roles of cGAS-STING in CRC

Tumor-promoting inflammation is a critical enabling characteristic of cancer (5), and the cGAS-STING pathway plays a vital role in both inflammatory progression and the inflammation-to-cancer transition. In colitic tissues, cGAS-STING signaling is markedly activated (85), accompanied by aberrant STING protein accumulation (86). Treating murine colitis models with STING agonists exacerbates intestinal inflammation (87, 88), whereas cGAS-STING inhibition alleviates disease severity (89, 90).

Beyond endogenous DNA damage, gut microbiota dysbiosis is another major driver of persistent STING activation (86). Specifically, dysbiotic microbes and their metabolites induce K63-linked ubiquitination of STING in myeloid cells, enhancing its stability and aggravating colitis (86). Conversely, fecal microbiota transplantation (FMT) reverses this aberrant accumulation and ameliorates inflammation in a STING-dependent manner (86, 91). This highlights a “dysbiosis-persistent STING activation” axis that continuously drives inflammatory progression.

Excessive cGAS-STING activation during inflammation usually promotes intestinal epithelial cell apoptosis and impairs barrier integrity (87). During repeated cycles of chronic injury and regeneration, intestinal epithelial cells (IECs) progressively accumulate mutations, eventually leading to tumorigenesis. Concurrently, the sustained release of STING-downstream inflammatory factors recruits abundant immune cells and polarizes macrophages and Th cells toward pro-inflammatory phenotypes. This maintains chronic inflammation, exacerbates epithelial damage, and fosters a tumor-supportive microenvironment (74, 92). Accordingly, genetic deletion or pharmacological inhibition of STING reduces colon tumor formation in inflammation-driven mouse models (93–95). Interestingly, the expression of interferon-gamma inducible protein 16 (IFI16), a cGAS-STING-related protein, declines during the colitis-to-cancer transition (96), suggesting a potential functional shift in cGAS-STING signaling during this process.

Notably, even after tumor formation, chronic STING activation can facilitate cancer progression. In cancer cells with high chromosomal instability (CIN), persistent STING activation redirects downstream signaling from IFN-I production to the non-canonical NF-κβ cascade, promoting invasion and metastasis (97). This mechanism is conceivable to be particularly relevant to CIN-positive, MSS CRC tumors. Furthermore, prolonged IFN-I release can upregulate PD-L1 and indoleamine 2,3-dioxygenase (IDO) on tumor cells, potentially causing effector T-cell exhaustion (98). In MC38 tumor models, STING hyperactivation has also been shown to induce T-cell death (99). Additionally, persistent STING activation drives the expansion of regulatory B cells (Bregs), which secrete inhibitory cytokine IL-35 to suppress natural killer (NK) cell proliferation and anti-tumor responses (100). Under certain conditions, tumor-derived inflammatory factors may also recruit MDSCs to establish a localized immunosuppressive network.

In summary, the role of cGAS-STING in CRC is highly context-dependent. During early tumorigenesis, cGAS-STING often triggers tumor-promoting inflammation. Its sustained hyperactivation can foster immunosuppression. Conversely, acute or properly calibrated activation initiates anti-tumor immunity. Therefore, precisely modulating the cGAS-STING pathway to maximize its anti-tumor functions while minimizing pro-tumorigenic effects remains a critical challenge for future therapeutic development.

## 4 Crosstalk between cGAS-STING pathway and lipid metabolism

Tumor metabolic state profoundly shapes immune signaling and effector functions within TME (101). As a key component of metabolic reprogramming, lipid metabolism not only supports tumor growth and survival but also profoundly influences innate and adaptive immune responses (102). Emerging evidence indicates bidirectional crosstalk between lipid metabolic networks and the cGAS–STING pathway (103–105). In the colorectal microenvironment, this crosstalk is further diversified by gut microbiota-derived lipid metabolites. Given the context-dependent functions of cGAS–STING, lipid metabolism may represent a pivotal regulator of its pro-tumorigenic versus anti-tumorigenic effects. Therefore, we next summarize the interplay between lipid metabolism and cGAS–STING signaling across different diseases and discuss the underlying mechanisms. **Table 1** summarizes the evidence status of these mechanisms in CRC and non-CRC models.

**Table 1:** Evidence landscape and knowledge gaps in the lipid–cGAS–STING axis in CRC.

Mechanistic interaction	Evidence in non-CRC models	Evidence in CRC	Knowledge gaps in CRC
Lipid-dependent mtDNA release → cGAS–STING	1. Lipotoxicity/ROS: SFA overload; FAO/LDs inhibition; 2. Membrane lipid remodeling: PUFA enrichment; cardiolipin loss 3. Direct enzyme modulation	Preliminary Lipid metabolic reprogramming; ACADS regulation (106); Lovastatin-induced mitochondrial oxidative stress (81)	1. Role of lipotoxicity/ROS in mtDNA leakage 2. Impact of specific mitochondrial membrane lipid remodeling
Subcellular Lipid micro-environment → cGAS–STING	1.Plasma Membrane: PI(4,5)P_2_ (resting cGAS) 2. ER: Cholesterol (STING retention); MUFAs/PI(3,5)P_2_ (STING export) 3.Golgi: Cholesterol, sphingomyelin, PI(3,5)P_2_ (STING oligomerization) 4.Endosome: PI4P (STING polymerization)	Preliminary Significant alterations in cholesterol & phospholipid profiles; STING ER phase: Radiation-induced, increased HMGCR/cholesterol traps STING in ER (radioresistance) (107)	1. Unverified spatial impact of membrane phosphoinositides & lipid rafts on STING trafficking/oligomerization 2. How specific lipidome shifts quantitatively dictate cGAS-STING signaling intensity 3. Efficacy of targeting spatial lipid networks to rescue immunity
Lipid-associated PTMs → cGAS–STING	1. Palmitoylation: cGAS, STING 2. Inhibitory PTMs: Nitroalkylation (NO_2_-FAs); Carbonylation (4-HNE)	Limited Enhanced lipid synthesis and sufficient palmitoylation substrates; Clearance of 4-HNE by GPX4 maintains STING activation (carbonylation not verified) (108)	1. Crosstalk between lipid synthesis and specific PTMs 2. Roles of palmitoylation in the cGAS-STING pathway 3. Impact of CRC-specific lipid metabolites on STING palmitoylation/carbonylation
cGAS–STING → lipid metabolic homeostasis	1. Physiological: Maintains homeostasis 2. Low-intensity (Lipotoxic stress): Promotes lipid accumulation 3. Chronic/Robust: Accelerates catabolism & dysbiosis	Limited /	1. Validation of the 3-trajectory paradigm and their impacts in CRC progression
cGAS–STING → lipid peroxidation and ferroptosis	1. Ferroptosis drivers: Iron accumulation; antioxidant loss; mitochondrial ROS 2. Negative feedback and immune suppression	Validated Antitumor effect by acute, robust STING activation (109, 110)	1. Exact threshold defining beneficial ferroptosis vs. detrimental hyper-peroxidation
Microbiota-derived lipids → cGAS–STING	/	Validated DCA-mediated STING activation (111, 112); Context/dose-dependent “butyrate paradox” (113, 114)	1. Defining optimal therapeutic doses of microbiome-derived DCA/butyrate to avoid paradoxical immunosuppression and tune local STING tone

This table summarizes the mechanistic crosstalk between lipid metabolism and the cGAS-STING signaling pathway, categorizing the evidence observed in non-CRC models versus CRC models, and highlighting current knowledge gaps for future therapeutic translation. Evidence status in CRC was classified according to the strength of mechanistic support reported in the literature. “Validated” indicates direct mechanistic evidence in CRC models, “Preliminary” indicates that relevant components or partial causal links have been demonstrated in CRC, whereas “Limited” indicates indirect or insufficient CRC-specific evidence. Evidence from non-CRC models is considered hypothesis-generating and should not be interpreted as definitive CRC-specific evidence. 4-HNE, 4-hydroxynonenal; ACADS, short-chain acyl-CoA dehydrogenase; cGAS, cyclic GMP-AMP synthase; CRC, colorectal cancer; DCA, deoxycholic acid; ER, endoplasmic reticulum; FAO, fatty acid oxidation; GPX4, glutathione peroxidase 4; HMGCR, 3-hydroxy-3-methylglutaryl-CoA reductase; LDs, lipid droplets; mtDNA, mitochondrial DNA; MUFAs, monounsaturated fatty acids; NO_2_-FAs, nitro-fatty acids; PI(3,5)P_2_, phosphatidylinositol 3,5-bisphosphate; PI(4,5)P_2_, phosphatidylinositol 4,5-bisphosphate; PI4P, phosphatidylinositol 4-phosphate; PTMs, post-translational modifications; PUFA, polyunsaturated fatty acid; ROS, reactive oxygen species; SFA, saturated fatty acid; STING, stimulator of interferon genes.

### 4.1 Regulation of cGAS-STING pathway by lipid metabolic reprogramming

Tumor-intrinsic lipid reprogramming alters intracellular lipid profiles. This can remodel local microenvironments and substrate pools while frequently inducing lipotoxicity under metabolic stress. Consequently, lipid reprogramming may exert a multidimensional impact on the cGAS-STING pathway. Current evidence suggests three primary regulatory mechanisms: driving mtDNA release, directing the subcellular localization and transport of pathway components, and modulating lipid-associated post-translational modifications (**Figures 2**, **3**).

**Figure 2:**
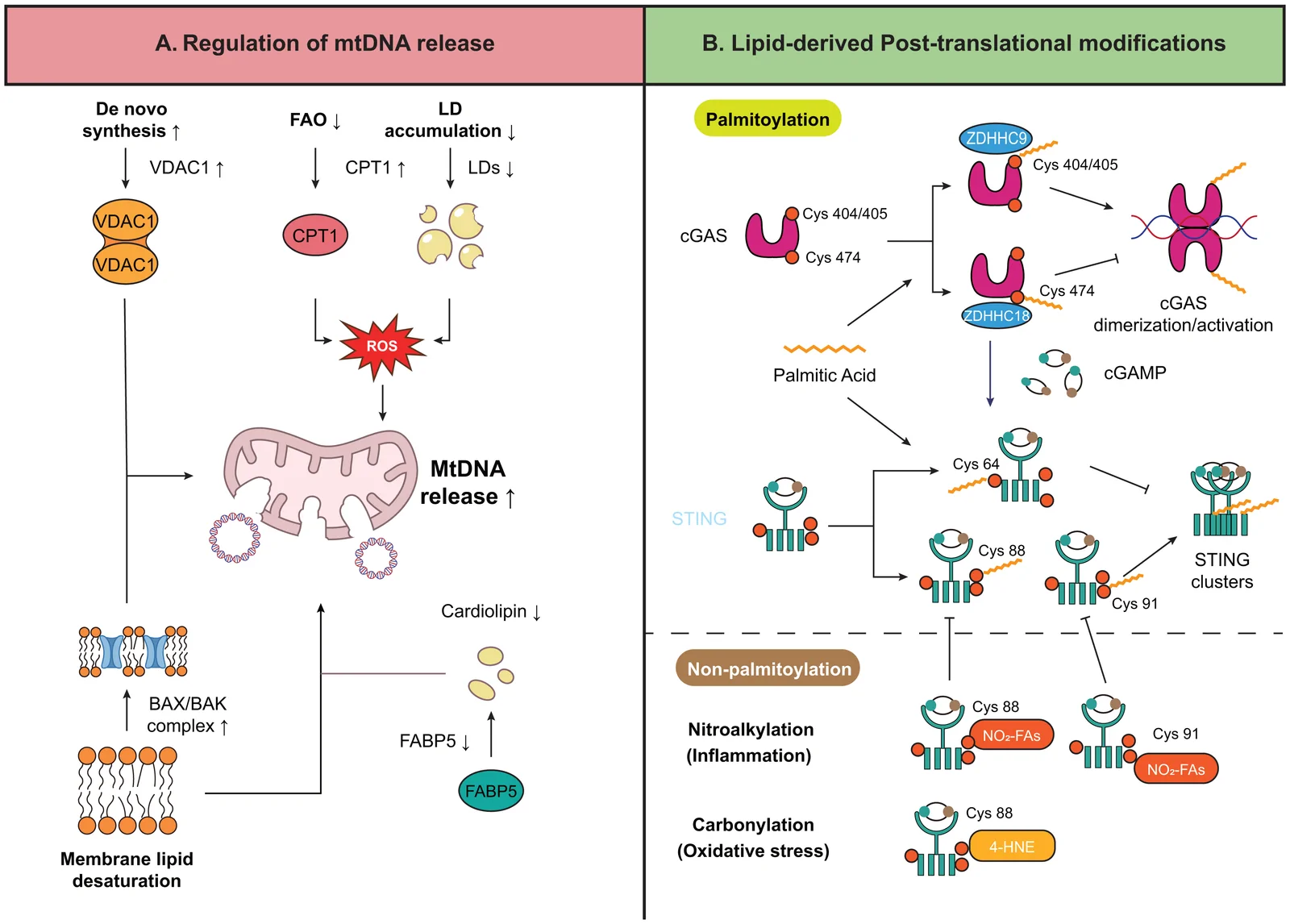
Regulation of mtDNA release and lipid-derived post-translational modifications of cGAS and STING. **(A)** Regulation of mtDNA release. Lipid dysregulation triggers cytosolic mtDNA leakage through multiple pathways. Enhanced *de novo* synthesis has been reported to promote VDAC1 oligomerization. Reduced FAO (via CPT1 inhibition) and disrupted LD accumulation can contribute to ROS-mediated mitochondrial damage. Membrane lipid desaturation can enhance BAX/BAK pore-forming activity, and FABP5 inhibition impairs cardiolipin synthesis, both of which accelerate mtDNA release into the cytosol. **(B)** Lipid-derived post-translational modifications. Lipid metabolites directly modulate cGAS-STING activity. Palmitoylation by ZDHHCs exerts site-specific effects: it activates cGAS (Cys404/405) and STING (Cys88/91, promoting clustering), but suppresses cGAS (Cys474) and maintains STING in an inactive state (Cys64). Non-palmitoylation modifications, including nitroalkylation by NO_2_-FAs (Cys88/91) during inflammation and carbonylation by 4-HNE (Cys88) under oxidative stress, inhibit STING activation. mtDNA, mitochondrial DNA; FAO, fatty acid oxidation; CPT1, carnitine palmitoyltransferase 1; LD, lipid droplet; ROS, reactive oxygen species; VDAC1, voltage-dependent anion channel 1; FABP5, fatty acid-binding protein 5; cGAS, cyclic GMP-AMP synthase; STING, stimulator of interferon genes; cGAMP, cyclic guanosine monophosphate–adenosine monophosphate; ZDHHC, zinc finger DHHC-type palmitoyltransferase; Cys, cysteine; NO_2_-FAs, nitro-fatty acids; 4-HNE, 4-hydroxynonenal.

**Figure 3:**
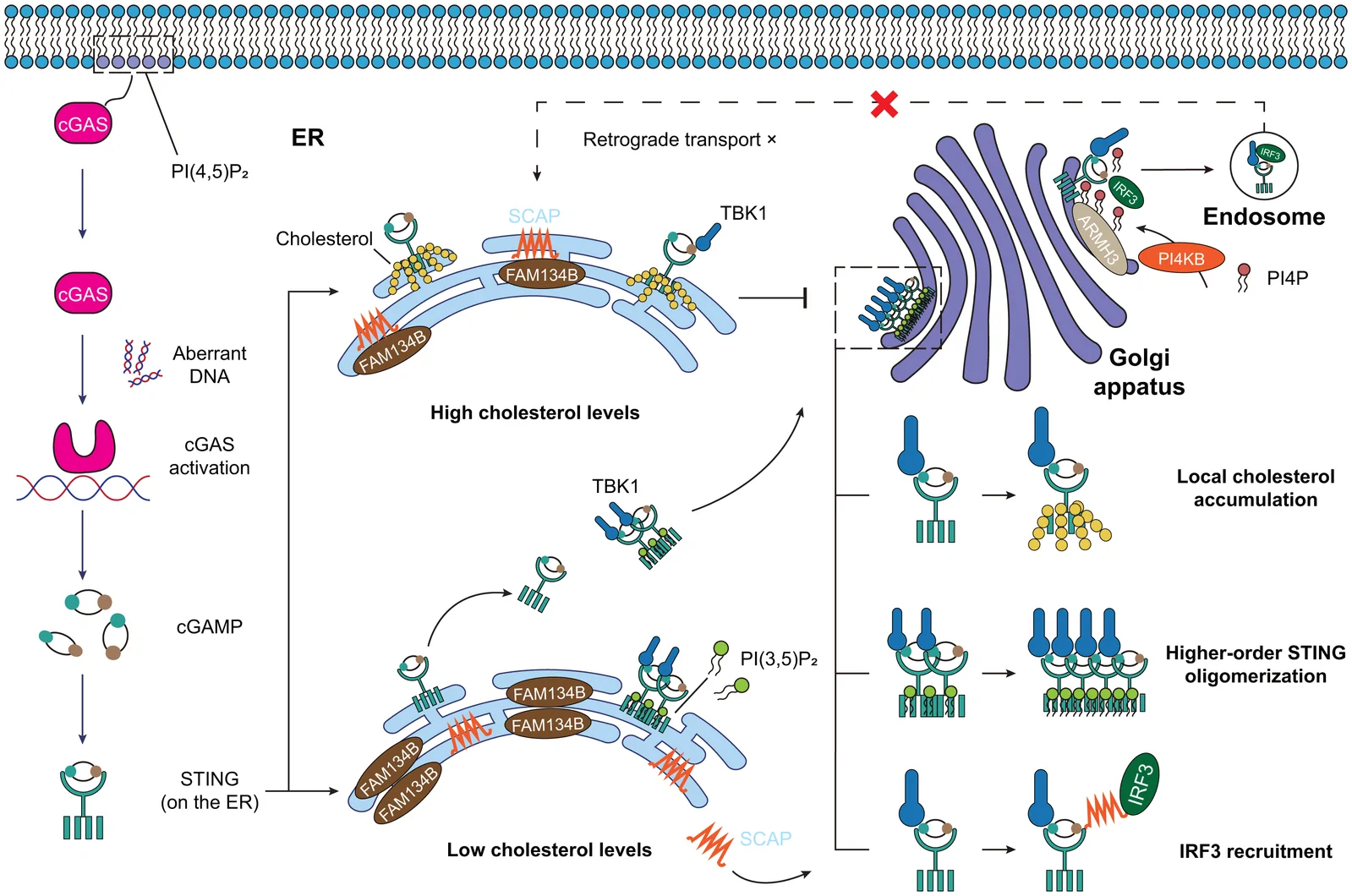
Lipid microenvironment regulates cGAS-STING subcellular localization, trafficking, and aggregation. cGAS is anchored to the plasma membrane by PI(4,5)P_2_ in the resting state. Upon sensing aberrant DNA, cGAS dissociates and synthesizes cGAMP, which activates ER-resident STING. At the ER, local cholesterol levels dictate STING trafficking. High cholesterol retains STING through direct binding and the formation of a SCAP-FAM134B quiescent complex. Conversely, low cholesterol relieves this inhibition. PI(3,5)P_2_ can promote STING oligomerization, while FAM134B facilitates STING trafficking from the ER to the Golgi. At the Golgi membrane, local cholesterol accumulation and PI(3,5)P_2_ binding promote higher-order STING oligomerization, while ER-released SCAP serves as a scaffold to recruit IRF3. Subsequently, ARMH3 recruits PI4KB to generate PI4P, which directs STING trafficking to endosomes and prevents retrograde transport back to the ER. PI(4,5)P_2_, phosphatidylinositol 4,5-bisphosphate; cGAS, cyclic GMP-AMP synthase; cGAMP, cyclic guanosine monophosphate–adenosine monophosphate; ER, endoplasmic reticulum; STING, stimulator of interferon genes; SCAP, SREBP cleavage-activating protein; PI(3,5)P_2_, phosphatidylinositol-3,5-bisphosphate; TBK1, TANK-binding kinase 1; IRF3, interferon regulatory factor 3; ARMH3, Armadillo-like helical domain-containing protein 3; PI4KB, phosphatidylinositol 4-kinase beta; PI4P, phosphatidylinositol 4-phosphate.

#### 4.1.1 Lipid dysregulation promotes mtDNA release

Due to its distinctive structural features and higher copy number, mtDNA is more readily accessible for immune sensing than nuclear DNA and is also more vulnerable to damage in cancers (115). As a result, mtDNA functions as a key endogenous activator of the cGAS-STING pathway (54). Generally, mtDNA is released into the cytoplasm through the mPTP, VDAC or BAX/BAK channels, following changes in mitochondrial inner membrane permeability caused by defects in mitochondrial replication or transcription machinery or by the accumulation of ROS (116).

Lipotoxicity from lipid overload is a major trigger for cytoplasmic mtDNA release. Under metabolic stress, the accumulation of SFAs, such as palmitic acid, can induce mitochondrial dysfunction and ROS production, increasing mitochondrial membrane permeability and subsequent mtDNA release, thereby activating cGAS and its downstream signaling (117–119). When cellular biochemical processes associated with lipid accumulation undergo aberrant reprogramming, opportunities for mtDNA leakage often increase. For instance, inhibition of CPT1A leads to intracellular lipid accumulation and elevated ROS levels. The resulting mitochondrial damage is sufficient to activate the cGAS-STING pathway (120). Enhanced fatty acid synthesis can mediate the oligomerization of VDAC1 to form channels, promoting mtDNA release and activating the inflammatory response via the cGAS-STING pathway (121). Similarly, LDs can protect mitochondria from lipotoxic damage (36), whereas targeting LDs can induce oxidative stress in tumor cells (122, 123). Disruption of LD synthesis or promotion of their degradation may activate the cGAS-STING pathway by damaging mitochondria and causing mtDNA leakage. Specifically, lipid metabolic enzymes in CRC can modulate mtDNA dynamics via non-lipotoxic pathways: short-chain acyl-CoA dehydrogenase (ACADS) deficiency suppresses mtDNA leakage by promoting mtDNA methylation, whereas its activation reactivates mtDNA release and triggers antitumor immunity (106).

Changes in mitochondrial membrane lipid composition are also a critical factor. On one hand, when mitochondrial membrane lipids are remodeled to contain highly unsaturated species during ROS accumulation, the membrane becomes more susceptible to oxidation, impairing mitochondrial function. In certain cells, high PUFA content in the mitochondrial membrane predisposes mitochondria to rupture and release mtDNA, which can activate the cGAS-STING pathway in surrounding macrophages (124). A membrane environment enriched with unsaturated lipids also promotes the pore-forming activity of the pro-apoptotic BAX/BAK complex, accelerating mtDNA release into the cytosol and subsequent cGAS activation (125). Notably, excessive lipid peroxidation generates lipid peroxides such as 4-hydroxynonenal (4-HNE), which instead impairs STING activation in CRC (108). Hence, the effect of lipid peroxidation on STING signaling is dose-dependent. On the other hand, interfering with mitochondrial membrane lipid content can compromise mitochondrial stability and promote mtDNA release. Inhibition of FABP5 impairs the synthesis of cardiolipin, an essential mitochondrial membrane lipid, leading to a punctate mitochondrial morphology and mtDNA release, which activates cGAS (126). Targeting mitochondrial membrane lipid remodeling may represent a potential strategy for activating the cGAS-STING pathway.

#### 4.1.2 Lipid microenvironment regulates cGAS-STING subcellular localization, trafficking, and aggregation

The activation of the cGAS-STING signaling pathway depends on the precise localization of key proteins, a process often linked to lipids (**Figure 3**). For example, the N-terminal domain of cGAS binds to the plasma membrane lipid phosphatidylinositol 4,5-bisphosphate [PI(4,5)P_2_] in the resting state, preventing cGAS from accessing self-DNA and avoiding aberrant autoimmune responses (127). Only when cGAS dissociates from PI(4,5)P_2_ and is released to engage with appropriate stimuli are downstream immune effects activated. Redistribution of PI(4,5)P_2_ in tumor cells may lead to cGAS dysfunction.

As an ER-resident protein, STING activation largely relies on its precise trafficking from the ER to the Golgi apparatus, a process partly regulated by intracellular lipids and lipid metabolism. At the ER stage, local lipid concentrations participate in regulating STING retention and release. ER cholesterol levels act as a retention signal for STING. The transmembrane domain of STING contains a specific cholesterol-binding motif (CRAC). ER cholesterol directly binds to this motif, limiting ER membrane curvature and physically retaining STING within the ER (51). Elevated cholesterol levels induce SREBP cleavage-activating protein (SCAP) to form an ER membrane-anchored complex with the autophagy receptor FAM134B, which also maintains STING in a quiescent state (128). Upon cholesterol depletion, STING is released from ER retention and spontaneously traffics to the Golgi apparatus (51, 129, 130). Meanwhile, FAM134B released from the SCAP–FAM134B complex cooperates with COPII vesicles to facilitate STING export from the ER (128). Conversely, SCD1-generated MUFAs act as a biophysical switch; by increasing ER membrane curvature and fluidity, MUFAs counteract retention constraints and license STING conformational release (131). Furthermore, basal levels of phosphatidylinositol-3,5-bisphosphate [PI(3,5)P_2_] in the ER serve as an essential lipid ligand that supports the initial trafficking of STING, and impaired PI(3,5)P_2_ binding results in transport defects (132).

Following its translocation to the Golgi apparatus, the lipid microenvironment becomes a key determinant of STING signal activation. STING requires cholesterol- and sphingomyelin-enriched lipid raft domains within the Golgi membrane to support its phosphorylation (133). Following palmitoylation, STING orchestrates local cholesterol enrichment on the cytosolic leaflet of the membrane, thereby forming higher-order signaling clusters that facilitate kinase recruitment (134). Cryo-electron microscopy research further revealed that PI(3,5)P_2_ and cholesterol are directly incorporated into the interface between two STING dimers, functioning as a molecular glue that promotes stable higher-order oligomerization. This structural organization provides a favorable structure platform for TBK1 activation (132, 135). Concurrently, SCAP, released from the ER, functions as a physical scaffold during trafficking. Its N- and C-terminal domains bind STING and IRF3, respectively, thereby recruiting IRF3 and enabling efficient TBK1-mediated phosphorylation of IRF3 (136).

During subsequent trafficking to the trans-Golgi network and endosomal compartments, lipid metabolism further promotes signal assembly and amplification. Golgi-localized Armadillo-like helical domain-containing protein 3 (ARMH3) recruits phosphatidylinositol 4-kinase beta (PI4KB) to generate phosphatidylinositol 4-phosphate (PI4P). This process directs STING trafficking toward acidic endosomal compartments, hence enhancing STING polymerization and preventing its retrograde transport back to the ER (137).

In conclusion, lipid molecules and their associated sensing proteins exert highly spatially dependent control throughout STING trafficking. In particular, high cholesterol levels in the ER suppress early STING trafficking through direct binding and maintenance of retention complexes, whereas cholesterol in the Golgi promotes STING palmitoylation and downstream signaling amplification. The distinct subcellular effects of the same lipid demonstrate that it modulates STING beyond a simple linear model, ensuring precise regulation of innate immune signaling.

#### 4.1.3 Lipid-associated post-translational modifications alter cGAS-STING activity

Post-translational modifications (PTMs) are a critical mechanism for regulating cGAS-STING pathway activity (138). Intermediate metabolites generated through lipid metabolic reprogramming can directly participate in these PTMs to modulate cGAS-STING activity. Among lipid-related PTMs, palmitoylation represents a key lipid modification mechanism. Mediated by zinc finger Aspartate-Histidine-Histidine-Cysteine-type palmitoyltransferases (ZDHHCs), this modification attaches palmitic acid to cysteine residues of target proteins via a thioester bond, thus regulating signal transduction (139).

Both cGAS and STING are regulated by palmitoylation (**Figure 2B**). Palmitoylation of cGAS at cysteines 404/405 (Cys404/405) by ZDHHC9 promotes its dimerization and activation (140), whereas ZDHHC18-mediated palmitoylation at Cys474 suppresses DNA binding and serves as a negative feedback mechanism following dsDNA recognition (141). For STING, palmitoylation is essential for its activation. After translocating to the Golgi apparatus, STING undergoes palmitoylation at Cys88 and Cys91 catalyzed by ZDHHCs, a modification required for TBK1 recruitment (8). As two TBK1 dimers must come into close proximity for mutual phosphorylation, STING must form clusters to increase contact opportunities. This explains why single-molecule localization microscopy reveals STING clusters of approximately 20 molecules on the Golgi apparatus, with individual molecules interacting via palmitoylation (134). When these residues are mutated or pharmacologically blocked, STING palmitoylation is prevented, hindering its clustering on the Golgi apparatus and reducing IFN secretion (8, 9, 142). In contrast, palmitoylation at Cys64 appears to maintain STING in an inactive state under basal conditions, indicating the site-specific effects of this modification (143). Lipid metabolism also influences STING palmitoylation. When FASN activity is inhibited, malonyl-CoA accumulates in the cell and binds to the Cys91 residue of STING, blocking its palmitoylation (144).

Other lipid-derived PTMs negatively regulate STING activity. Nitro-fatty acids (NO_2_-FAs) generated during inflammation can undergo nitroalkylation with STING at Cys88/91 and at an N-terminal histidine (His16), directly blocking its palmitoylation and inhibiting IFN release (145). Similarly, 4-HNE, a lipid peroxidation product generated during oxidative stress, can mediate carbonylation at the Cys88 residue of STING, which inhibits STING translocation to the Golgi apparatus (146). Deficiency of Autophagy-related protein 16-like 1 (ATG16L1) in macrophages reduces 4-HNE production, paradoxically strengthening STING pathway activity (147). Unlike the reversible nature of palmitoylation, nitroalkylation and carbonylation cause irreversible STING inactivation.

### 4.2 Regulation of lipid metabolism reprogramming by cGAS-STING pathway

In response to metabolic stress, the activated cGAS-STING pathway not only mediates immune responses but also directly reshapes cellular lipid metabolism through non-canonical functions, forming complex feedback loops.

#### 4.2.1 cGAS-STING pathway influences lipid metabolic homeostasis

Accumulating evidence indicates that STING, along with its upstream and downstream key proteins, directly or indirectly participates in lipid metabolic pathways. cGAS has been found to directly bind to and inhibit aldehyde dehydrogenase 2 (ALDH2), thereby reducing acetyl-CoA production and suppressing lipogenic gene transcription (148). STING itself is also extensively implicated in lipid metabolic regulation. Studies in *Drosophila melanogaster* have shown that STING localized on the ER forms a multienzyme complex with ACC and FASN, promoting lipid synthesis (149). When STING is inactivated, both fatty acid synthesis and lipid storage are significantly reduced in *Drosophila* cells. Concurrently, malonyl-CoA accumulation inhibits CPT1 activity, suppressing FAO and rendering cells more reliant on glucose metabolism for energy (149). These findings suggest that the primordial function of STING may involve the regulation of lipid metabolic homeostasis. Mammalian model studies have shown that STING knockout under physiological conditions leads to increased fat accumulation and obesity in mice (150). Mechanistic investigations reveal that STING can localize directly to mitochondria, promoting CPT1A degradation and thereby suppressing excessive FAO in tumor cells and maintaining systemic lipid homeostasis (151). Additionally, IRF3-deficient mice develop obesity, insulin resistance, and type 2 diabetes (152). Taken together, these insights highlight the pivotal involvement of cGAS-STING in regulating lipid metabolic homeostasis.

However, the effect of STING on lipid metabolism appears to be context-dependent. Specifically, under high-fat metabolic stress, low-intensity STING activation induced by lipotoxicity tends to suppress lipid consumption and promote accumulation. In a nonalcoholic fatty liver disease (NAFLD) model, STING1 recruits and activates mTORC1 via the scaffold protein SQSTM1, thereby inhibiting hepatocyte lipophagy and leading to lipid accumulation (117). In macrophages, STING suppresses yes-associated protein (YAP) to block fusion between autophagosomes and LDs, which subsequently drives hepatic lipid accumulation (153). Consistently, under high-fat diet conditions, *Sting^gt/gt^* mice, in which downstream STING signaling is blocked, exhibit attenuated metabolic effects including reduced obesity, lower circulating lipid levels, and alleviated steatosis (154–156). Conversely, when the cGAS-STING pathway undergoes robust and sustained activation, its metabolic regulatory orientation is reversed. For instance, in a mouse model of autoimmune disease, chronic STING activation leads to sustained TBK1 activation, which inhibits mTORC1 activity, thereby accelerating cellular lipid catabolism rather than promoting lipid accumulation (157). Furthermore, chronic STING activation impairs the production of metabolically protective PUFAs by inhibiting FADS2, further exacerbating dyslipidemia (158). Therefore, STING appears to maintain lipid metabolic homeostasis under physiological conditions, whereas its chronic activation can amplify metabolic disorders. Notably, signaling intensity and cellular context may shape the trajectory of lipid metabolic dysregulation, the precise mechanisms of which warrant further investigation.

#### 4.2.2 cGAS-STING pathway induces lipid peroxidation and ferroptosis

Another key metabolic consequence of cGAS-STING activation is the induction of ferroptosis. Ferroptosis is a non-apoptotic form of cell death driven by iron accumulation and membrane lipid peroxidation (159). Elevated intracellular iron levels, accumulation of oxidizable lipids, or dysregulation of the intrinsic redox system can trigger membrane lipid peroxidation, ultimately leading to ferroptosis (159). IFN-β released via cGAS-STING signaling not only enhances immunity but also increases ferroptosis sensitivity (160). Components of the cGAS-STING pathway can further modulate ferroptosis susceptibility by altering intracellular iron concentrations and antioxidant levels. In disease models such as atherosclerosis (161) and acute kidney injury (162), cGAS-STING has been shown to upregulate nuclear receptor coactivator 4 (NCOA4), induce ferritinophagy, and release free iron into the cytoplasm, culminating in lipid peroxidation and ferroptosis. STING can also activate signal transducer and activator of transcription 3 (STAT3), promoting autophagic degradation of ferritin (163). On the antioxidant front, STING activation triggers autophagic degradation of the antioxidant enzyme glutathione peroxidase 4 (GPX4), exacerbating oxidative stress-mediated lipid peroxidation and ferroptosis (164, 165). Under oxidative stress, ER-localized STING translocates to mitochondria to promote mitochondrial fusion, which generates a burst of ROS leading to ferroptosis (166). In CRC, acute and robust STING activation has been demonstrated to induce tumor cell ferroptosis in preclinical models, further enhancing antitumor efficacy (109, 110).

However, intense lipid peroxidation mediated by hyperactivated STING also confers severe cytotoxicity to normal IECs and immune cells (167). Concurrently, STING pathway activation promotes the generation of peroxidation products such as 4-HNE (167). Accumulated 4-HNE, in turn, suppresses STING activation and translocation through carbonylation (146), establishing a negative feedback loop for the STING pathway. Furthermore, hyperactive lipid peroxidation generates substantial amounts of immunosuppressive mediators, such as prostaglandin E2 (PGE2), which ultimately dampens the antitumor immune response. Taken together, these findings demonstrate that STING-mediated lipid peroxidation is not inherently beneficial at maximum intensity; rather, its ultimate functional output is tightly constrained by the duration and magnitude of signal activation.

### 4.3 Gut ecology and lipid–cGAS-STING crosstalk in CRC

Distinct from universally conserved mechanisms, lipid-cGAS–STING crosstalk in CRC is uniquely shaped by the gut microenvironment. Metabolites derived from the colonizing microbiota contribute to the intestinal immune landscape. Prominent among these are lipid-derived metabolites, notably secondary bile acids and short-chain fatty acids (SCFAs), which participate in balancing intestinal inflammation and tumor immunity by modulating immune cascades, including the cGAS-STING pathway.

Secondary bile acids are host-microbiota cometabolites formed in the gut via the microbial biotransformation of liver-synthesized primary bile acids. Deoxycholic acid (DCA), a predominant secondary bile acid in humans, is primarily generated by bacteria such as *Clostridium scindens* (167). In CRC, microbiota-derived DCA is significantly elevated, and high concentrations of DCA have been widely recognized as a pro-tumorigenic driver (168, 169). Mechanistically, DCA induces lipid peroxidation and ferroptosis in IECs by activating hypoxia-inducible factor-2α and divalent metal transporter-1, compromising intestinal barrier integrity (170). Concurrent exogenous DCA supplementation exacerbates CRC progression by inducing DNA damage in intestinal stem cells (171). Given that both lipid peroxidation and aberrant DNA release are critical triggers for cGAS-STING activation, these observations align with direct links between DCA and STING activation. Indeed, DCA can induce the formation of mitochondrial Bax/Bak pores and the subsequent mtDNA release and STING signaling activation (111). Furthermore, DCA has been demonstrated to bind the transmembrane domain of STING, promoting STING oligomerization (112). Taken together, microbiota-derived DCA appears to exert a dose-dependent dual effect: whereas high concentrations accelerate tumor progression, low concentrations may elicit anti-tumor immunity by triggering the cGAS-STING pathway.

SCFAs, predominantly produced through the fermentation of dietary fibers by intestinal anaerobes including *Bacteroides* and *Clostridium*, act as protective metabolites deeply integrated into host immune regulation and metabolic crosstalk (172). Butyrate serves as the prototypical SCFA. In inflammatory bowel disease models, butyrate has been shown to attenuate STING phosphorylation, subsequently mitigating ER stress or pyroptosis in IECs to resolve intestinal inflammation (173, 174). In the oncological context, clinical evidence reveals a substantial depletion of both fecal butyrate and butyrate-producing bacteria in patients with advanced CRC compared with healthy individuals, reinforcing its supportive role in preserving intestinal barrier integrity (175). Butyrate suppresses tumorigenesis via diverse, multifaceted mechanisms (176). Notably, as a natural histone deacetylase inhibitor (HDACi), butyrate promotes chromatin relaxation and provokes a DNA damage response. This process triggers a modest cGAS-STING signal, selectively upregulates MHC-I expression on tumor cells, and augments CD8^+^ T cell infiltration and immune recognition (113). However, excessively high butyrate concentrations can dampen the efficacy of immunotherapy (177). Butyrate can also induce radioresistance in CRC by inhibiting the phosphorylation of downstream TBK1/IRF3 within dendritic cells (114). This phenomenon likely occurs because the tissue microenvironment remodeled by anti-tumor therapies functionally mimics an acute inflammatory state, where elevated butyrate preferentially exerts its potent anti-inflammatory and immunosuppressive properties. Collectively, these studies encapsulate the “butyrate paradox”. On one hand, butyrate restrains hyperinflammation in inflammatory milieus while maintaining barrier integrity and conferring tumor suppression in neoplastic settings; on the other hand, different butyrate concentrations can orchestrate starkly contrasting phenotypic outcomes across different cell types like IECs versus macrophages (178). Therefore, it is imperative to delineate the intricate crosstalk between SCFAs and immune sensors like the cGAS-STING pathway, and to elucidate their distinct downstream mechanisms to rationally guide immunotherapeutic strategies for CRC.

## 5 Metabolic-immunotherapeutic strategies

For tumors characterized by dysregulated lipid metabolic reprogramming, particularly CRC, targeting this process or integrating it with interventions aimed at the cGAS-STING pathway may help convert tumors from “cold” to “hot”, thereby improving immunotherapy outcomes.

### 5.1 Targeting cholesterol metabolism and cGAS-STING pathway

Inhibiting cholesterol synthesis has been shown to activate the cGAS-STING pathway and induce IFN-I expression (129). Statins, classic inhibitors of HMG-CoA reductase, suppress cholesterol synthesis and have been associated with improved overall survival in cancer patients receiving ICIs, representing promising metabolic-immunomodulatory agents (179–181). Statins can relieve cholesterol-mediated blockade of STING translocation from the ER to the Golgi apparatus, restoring CD8^+^ T cell infiltration when combined with radiotherapy and overcoming radioresistance (107). Statin-mediated inhibition of cholesterol biosynthesis can lead to mitochondrial ROS accumulation and reduced mitochondrial membrane potential. This, in turn, induces mtDNA leakage, activates the cGAS-STING pathway, and triggers apoptosis (81). Nevertheless, statins may transiently suppress the IRF3 signaling axis by blocking the mevalonate pathway in immune cells (182). Thus, clinical application of statins requires careful optimization of administration strategies to balance their complex effects within the TME.

In addition to inhibiting intracellular cholesterol synthesis, strategies that deplete membrane cholesterol have shown preclinical promise in enhancing the cGAS-STING pathway and improving immunotherapy outcomes. These interventions alter the physical properties of the cell membrane. Increased permeability of the tumor cell membrane facilitates the entry and retention of anticancer drugs, thereby mitigating chemoresistance (183, 184). Concurrently, the actin cytoskeleton beneath the cell membrane undergoes remodeling and densification, increasing the overall mechanical stiffness of tumor cells. This mechanical change promotes the formation of stable immune synapses by T cells and enhances their cytotoxic capacity (185, 186). Depletion of membrane cholesterol also induces macrophage M1 polarization by disrupting lipid raft-mediated signal transduction (187). In parallel, hydrogen peroxide (H_2_O_2_) generated during cholesterol oxidation can be further converted into ROS, laying the foundation for oxidative stress-mediated STING activation (183, 187–189).

### 5.2 Inducing lipid peroxidation and ferroptosis to enhance cGAS-STING signaling

Inducing lipid peroxidation in tumor cells to activate the cGAS-STING signaling pathway represents another viable strategy. One prominent mechanism involves lipid peroxidation triggering mitochondrial membrane rupture and the subsequent release of mtDNA, ultimately leading to IFN-I release and immune activation (190, 191). The released IFNs can further promote ferroptosis in tumor cells, which is proposed to form a positive feedback loop that enhances antitumor immunity (190).

Disrupting antioxidant defenses, such as GPX4 and glutathione (GSH), is a core strategy for inducing lipid peroxidation. For instance, combining the BCL-2 inhibitor Sonrotoclax with radiotherapy downregulates GPX4 expression, thereby inducing ferroptosis and immune activation in tumor cells (192). Several nanoplatforms loaded with GPX4 inhibitors induce lipid peroxidation in the TME, releasing mtDNA to activate the cGAS-STING signaling pathway (193, 194). Moreover, certain nanoparticles utilize the valence states of loaded metal ions—including manganese (191, 195, 196), copper (197), molybdenum (196) and iridium (198) —to deplete intracellular GSH and disrupt the redox balance in tumor cells. Some of these metal-based compounds can also mimic multiple enzyme-like activities, achieving a multifactorial disruption of the antioxidant system (188, 199). Beyond targeting GSH and GPX4, interventions aimed at other proteins, such as MutT homolog 1 (MTH1) to impair the oxidative damage repair pathway, have also been demonstrated to activate STING (200).

Lipid peroxidation can also be induced by directly increasing ROS levels. Nanocarriers loaded with Brequinar or iridium complexes can trigger an intratumoral ROS burst *in situ*, switching on downstream cGAS-STING signaling (198, 201). Elevating intracellular labile iron levels or introducing catalytic metal ions can initiate the Fenton reaction within cells, converting hydrogen peroxide into the highly toxic hydroxyl radical (•OH). This directly damages lipids and promotes cGAS-STING activation (191, 197, 202–204). Additionally, a nanoplatform constructed to release calcium ions can induce mitochondrial calcium overload, indirectly causing a ROS burst in tumor cells (191, 195). Photodynamic therapy (PDT)-based photoimmunotherapy is an emerging treatment modality that utilizes photochemical reactions among light, oxygen, and photosensitizers to generate ROS. This approach induces lipid peroxidation in tumor cells, leading to ferroptosis and activation of the cGAS-STING pathway, hence potentiating anti-tumor immune responses (194, 198, 200, 205).

### 5.3 Nanodelivery strategies targeting lipid–cGAS-STING axis

As discussed above, CRC cells potentially construct barriers against innate immune activation through extensive lipid metabolic reprogramming. Though small-molecule drugs targeting these processes hold considerable promise for tumor therapy, their clinical translation is often hindered by systemic toxicity and limited efficacy. Statins, for example, can cause severe rhabdomyolysis (206). Lipid peroxidation-inducing drugs may also elicit off-target normal cell toxicity.

Recent advances in nanobiotechnology have provided a promising platform for constructing “lipid metabolism–immunity” cascade-targeting systems with improved bioavailability and potentially reduced off-target exposure. Emerging nanoplatforms incorporate metal ions to link lipid metabolic reprogramming with cGAS-STING activation. For instance, manganese-based nanozymes can participate in Fenton-like chemistry and exert enzyme-mimicking activities that deplete endogenous antioxidants. This process promotes lipid peroxidation and mtDNA leakage, thereby activating the cGAS-STING pathway (188). In addition, metal ions can act as important immune adjuvants for the anti-tumor function of this pathway, enhancing cGAS sensitivity to dsDNA (207) and cGAMP binding affinity to STING (184, 197, 208). Researchers have now developed “dual-effect” metabolic-immune nanoplatforms that both target cholesterol and induce lipid peroxidation, which can lead to higher antitumor efficacy. These advanced systems integrate cholesterol-depleting agents like cholesterol oxidase with transition metal-based nanozymes into a single functional unit (183, 187, 188). Once endocytosed, these nanoplatforms execute a multi-step cascade: first, they oxidize and deplete cell membrane cholesterol *in situ*. As detailed earlier, this depletion alters plasma membrane fluidity, destabilizes lipid raft architecture, and may alleviate STING retention within the ER. Simultaneously, the transition metals utilize the byproducts of cholesterol oxidation (e.g., H_2_O_2_) to catalyze Fenton-like reactions, generating elevated levels of ROS. This tailored ROS burst drives lipid peroxidation and disrupts mitochondrial integrity, leading to mtDNA leakage and anti-tumor immunity (183, 187, 188).

Overall, these studies highlight nanodelivery as an appealing strategy to couple metabolic disruption with innate immune activation. By selectively manipulating cholesterol homeostasis, lipid peroxidation, and STING signaling, nanoplatforms may transform metabolic vulnerabilities of CRC cells into spatially confined immunostimulatory signals. This mechanism-guided perspective also supports the broader concept that nanodelivery should be integrated with, rather than separated from, the lipid–cGAS-STING crosstalk.

## 6 Concluding remarks and future perspectives

Lipid metabolism and cGAS–STING signaling form a dynamic, highly context-dependent regulatory network across different cell types, a feature particularly evident in CRC. During the early stages of CRC development, oncogenic APC/β-catenin signaling promotes *de novo* lipogenesis, providing transformed intestinal epithelial cells with increased lipid availability for membrane biogenesis, energy production, and signal transduction. As tumors progress, CRC cells increasingly enhance CD36- and FABP-mediated lipid uptake, FAO, and lipid droplet formation to cope with immune pressure and nutrient deprivation. Meanwhile, *KRAS*-mutant CRC cells undergo sustained membrane lipid remodeling, which not only supports membrane homeostasis but also increases plasma membrane plasticity. These metabolic adaptations are accompanied by dynamic changes in cGAS–STING signaling. In early lesions, STING may undergo persistent but relatively low-level activation, contributing to a pro-tumor inflammatory milieu, whereas in advanced tumors, STING signaling is frequently suppressed to limit downstream antitumor immune responses. In parallel, progressive alterations in the gut microbiota and its metabolites, including DCA and butyrate, may further modulate cGAS–STING activity. These interconnected metabolic and immune programs shape the evolving tumor microenvironment throughout CRC progression.

Based on accumulating evidence, we propose that lipid metabolism may function as a potential checkpoint for cGAS–STING regulation in CRC. During early tumorigenesis, persistent aberrant activation of inflammatory pathways, including cGAS–STING, may promote intestinal epithelial injury and facilitate the acquisition of oncogenic alterations such as APC/β-catenin activation. These oncogenic programs may subsequently increase the expression and stability of fatty acid synthesis enzymes, including FASN and ACC, thereby expanding the intracellular fatty acid pool. These fatty acids may regulate STING signaling at multiple levels. For example, palmitate can serve as a substrate for STING palmitoylation, whereas *KRAS*-mutant-dependent, SCD-driven fatty acid desaturation may alter ER membrane fluidity, facilitating intracellular STING trafficking from the ER to the Golgi apparatus. Such metabolic–membrane coupling may help sustain STING activity and reinforce a chronic inflammatory and pro-tumor immune microenvironment.

With tumor progression, however, lipid metabolism may shift from supporting STING activation to constraining STING signaling. Increased cholesterol synthesis and cholesterol accumulation in the ER may promote STING retention in the membrane, limiting its trafficking and subsequent activation. This sterol-dependent retention is supported not only *in vivo* by radiation-induced HMGCR upregulation (107), but also structurally by the CRAC motif that physically coordinates membrane cholesterol (51). Meanwhile, enhanced FAO and lipid droplet formation can buffer lipotoxicity and preserve mitochondrial redox homeostasis, thus reducing mitochondrial damage and subsequent mtDNA leakage into the cytosol, which may in turn increase the threshold for cGAS activation. Consistently, the downregulation of specific lipid metabolic enzymes in CRC, such as ACADS, elicits a comparable immunosuppressive effect by stabilizing mtDNA and blunting its cytosolic leakage, underscoring a direct mechanistic link between metabolic dysregulation and STING signaling (106). Following membrane lipid remodeling, an appropriate MUFA/PUFA ratio may reduce the susceptibility of cancer cells to lipid peroxidation. Even when a fraction of membrane lipids undergoes peroxidation, the resulting products may enter the tumor microenvironment as immunosuppressive mediators or interact with STING to interfere with its palmitoylation and suppress downstream antitumor responses. Beyond tumor-intrinsic effects, a lipid-rich tumor microenvironment may induce metabolic dysfunction and functional exhaustion in infiltrating immune cells, further compromising effective antitumor immunity mediated by cGAS–STING signaling.

Conversely, these observations raise the possibility that appropriately timed and tuned activation of cGAS–STING may feed back to remodel tumor lipid metabolism. Transient but sufficiently strong STING stimulation may alter the metabolic dependencies of CRC cells, for example, by promoting CPT1A degradation to suppress FAO, modulating lipophagy, or increasing lipid peroxidation to induce ferroptosis. Such feedback may disrupt metabolic adaptations that support tumor cell survival while simultaneously enhancing antitumor immunity. Thus, lipid metabolism and cGAS–STING signaling are likely linked through a bidirectional regulatory relationship, whose functional consequences depend on tumor stage, cellular context, lipid composition, and the magnitude and duration of STING signaling.

This metabolic–immune coupling may be particularly relevant to MSS CRC, which accounts for most CRC cases. Compared with MSI CRC, MSS CRC may exhibit greater capacity for lipid uptake and lipid droplet formation (24). MSS CRC is also characterized by extensive membrane lipid remodeling and lipid raft enrichment, which may contribute to immune evasion (48). Persistent oxidative stress may further promote lipid peroxidation and aberrant accumulation of oxidized low-density lipoprotein (oxLDL), contributing to a lipid-rich immunosuppressive tumor microenvironment (209). These features raise the possibility that lipid metabolism and cGAS–STING signaling may be more tightly coupled in MSS CRC. However, direct studies of lipid metabolic programs in MSS CRC remain limited, and our current understanding is derived largely from studies of CRC as a whole. Systematically defining the lipidomic landscape of MSS CRC and determining how these metabolic features influence STING signaling will therefore be an important direction for future investigation.

Several key challenges remain before the lipid metabolism–cGAS–STING axis can be effectively translated into therapeutic strategies. First, given the context dependence of cGAS–STING signaling in CRC, future interventions should prioritize precise and titrated modulation rather than nonspecific, robust pathway activation. Particularly during early tumorigenesis, STING inhibition combined with appropriate metabolic intervention may be more therapeutically beneficial than sustained STING activation and may help alleviate chronic inflammation and T cell dysfunction (210). Second, metabolic manipulation of cGAS–STING will require sufficiently precise intervention to minimize systemic inflammatory toxicity. Stimulus-responsive delivery systems may enable spatiotemporal control of pathway activity (210). Mechanistically, metabolic interventions could be designed to preferentially promote mtDNA release rather than genomic DNA damage, or to modulate STING signaling through epigenetic mechanisms, as well as lipid-dependent control of STING oligomerization and membrane trafficking (211). Finally, targeted biomimetic delivery platforms may further restrict pathway modulation to the tumor microenvironment, reducing the systemic toxicity associated with conventional nanoparticles.

In summary, accumulating evidence supports a complex and bidirectional relationship between lipid metabolism and cGAS–STING signaling in CRC. We propose that lipid metabolism may represent a previously underappreciated metabolic checkpoint that influences CRC progression at different stages by regulating STING activation and downstream immune responses. Elucidating the molecular mechanisms underlying this metabolic–immune coupling, particularly in MSS CRC, may deepen our understanding of CRC immunometabolism and provide new opportunities to reprogram tumor metabolism and improve the efficacy and durability of cancer immunotherapy.

## Data Availability

The original contributions presented in the study are included in the article/supplementary material. Further inquiries can be directed to the corresponding author.

## Glossary

4-HNE: 4-hydroxynonenal
5-FU: 5-fluorouracil
ACC: Acetyl-CoA carboxylase
ACLY: ATP citrate lyase
ALDH2: Aldehyde dehydrogenase 2
ARMH3: Armadillo-like helical domain-containing protein 3
ATG16L1: Autophagy-related protein 16-like 1
ATM: Ataxia telangiectasia mutated
Bregs: Regulatory B cells
cGAS: Cyclic GMP-AMP synthase
cGAMP: Cyclic guanosine monophosphate–adenosine monophosphate
CIN: Chromosomal instability
CPT1A: Carnitine palmitoyltransferase 1A
CRAC: Cholesterol-binding motif
CRC: Colorectal cancer
DCs: Dendritic cells
dsDNA: Double-stranded DNA
EMT: Epithelial-mesenchymal transition
ER: Endoplasmic reticulum
FABPs: Fatty acid-binding proteins
FADS2: Fatty acid desaturase 2
FAO: Fatty acid oxidation
FASN: Fatty acid synthase
FFAs: Free fatty acids
FMT: Fecal microbiota transplantation
GPX4: Glutathione peroxidase 4
GSH: Glutathione
HDAC1: Histone deacetylase 1
ICI: Immune checkpoint inhibitor
IDO: Indoleamine 2,3-dioxygenase
IFI16: Interferon-gamma inducible protein 16
IFN-I: Type I interferons
IGFBP3: Insulin-like growth factor-binding protein
IL-22BP: IL-22 binding protein
IRF3: Interferon regulatory factor 3
LDs: Lipid droplets
MSI-H: Microsatellite instability-high
MSS: Microsatellite stable
mtDNA: Mitochondrial DNA
MTH1: MutT homolog 1
MUFAs: Monounsaturated fatty acids
NCOA4: Nuclear receptor coactivator 4
NF-κB: Nuclear factor κB
NK: Natural killer
NLRP3: NOD-like receptor protein 3
NO₂-FAs: Nitro-fatty acids
PDT: Photodynamic therapy
PI(3,5)P₂: Phosphatidylinositol-3,5-bisphosphate
PI(4,5)P₂: Phosphatidylinositol 4,5-bisphosphate
PI4KB: Phosphatidylinositol 4-kinase beta
PI4P: Phosphatidylinositol 4-phosphate
PLIN2: Perilipin 2
PREX2: Phosphatidylinositol-3,4,5-triphosphate-dependent Rac exchange factor 2
PTMs: Post-translational modifications
PUFAs: Polyunsaturated fatty acids
RNF183: Ring finger protein 183
ROS: Reactive oxygen species
SCAP: SREBP cleavage-activating protein
SCD1: Stearoyl-CoA desaturase 1
SFAs: Saturated fatty acids
SREBP1: Sterol regulatory element-binding protein 1
SREBP2: Sterol regulatory element-binding protein
STAT3: Signal transducer and activator of transcription 3
STING: Stimulator of interferon genes
TAMs: Tumor-associated macrophages
TBK1: TANK-binding kinase 1
TIME: Tumor immune microenvironment
TME: Tumor microenvironment
VCP: Valosin-containing protein
YAP: Yes-associated protein
ZDHHCs: Zinc finger Aspartate-Histidine-Histidine-Cysteine-type palmitoyltransferases
